# Toward Smart Salivary Diagnostics: A Comprehensive Review of Heavy Metal Biomarkers and Digital Risk Modeling

**DOI:** 10.3390/diagnostics16040635

**Published:** 2026-02-22

**Authors:** Claudia Florina Bogdan-Andreescu, Lucia Bubulac, Cristina-Crenguţa Albu, Dan Alexandru Slăvescu, Andreea Mariana Bănăţeanu, Oana Botoacă, Gabriela-Cornelia Muşat, Viorica Tudor, Emin Cadar, Mariana Păcurar

**Affiliations:** 1 Department of Specialty Disciplines, “Titu Maiorescu” University, 031593 Bucharest, Romania; claudia.andreescu@prof.utm.ro (C.F.B.-A.); andreea.banateanu@prof.utm.ro (A.M.B.); oana.botoaca@prof.utm.ro (O.B.); 2 Department of Family Medicine III, Faculty of Medicine, “Carol Davila” University of Medicine and Pharmacy, 050474 Bucharest, Romania; lucia.bubulac@umfcd.ro; 3 Department of Genetics, Faculty of Dentistry, “Carol Davila” University of Medicine and Pharmacy, 020021 Bucharest, Romania; 4 Department of Dentistry, Faculty of Medicine and Pharmacy, University of Oradea, 410073 Oradea, Romania; 5 Department of Otorhinolaryngology, Faculty of Dentistry, “Carol Davila” University of Medicine and Pharmacy, 020021 Bucharest, Romania; gabriela.musat@umfcd.ro; 6 Integrated Outpatient Department, “Prof. Dr. Agrippa Ionescu” Clinical Emergency Hospital, 011356 Bucharest, Romania; tudorviorica_med@yahoo.com; 7 Faculty of Pharmacy, “Ovidius” University, 900470 Constanța, Romania; emin.cadar@365.univ-ovidius.ro; 8 Department of Orthodontics, Faculty of Dental Medicine, George Emil Palade University of Medicine, Pharmacy, Science, and Technology of Târgu Mureș, 540139 Târgu Mureș, Romania; mariana.pacurar@umftgm.ro

**Keywords:** decision-support systems, diagnostic readiness levels, digital health diagnostics, exposure-informed biomarkers, oral–systemic interface, metallomics, precision dentistry, salivaomics, trace element analysis

## Abstract

**Background**: Saliva has been identified as a valuable diagnostic biofluid due to its non-invasive collection and its capacity to reflect oral and systemic biological processes. Advances in analytical chemistry, biosensing technologies, and artificial intelligence (AI)-assisted data integration have broadened the applications of salivary diagnostics. Among salivary exposome components, heavy metals such as lead, cadmium, mercury, nickel, chromium, arsenic, and aluminum serve as biologically and clinically relevant indicators of environmental exposure, toxic burden, and disease-associated molecular disorders. **Methods**: This structured review integrates clinical, experimental, and translational studies published between January 2020 and January 2026 that examined salivary heavy metal profiling in relation to oral health. Evidence was identified using systematic searches of PubMed/MEDLINE and supplementary sources. Studies were qualitatively assessed regarding analytical methodologies, reported concentration ranges, biological mechanisms, disease associations, and the development of digital and AI-assisted diagnostic applications. **Results**: Thirteen human clinical studies and six animal or in vivo investigations met the inclusion criteria. Across these studies, altered salivary metal profiles were linked to oxidative stress, inflammatory signaling, immune dysregulation, microbiome disturbances, and genotoxic markers relevant to periodontal disease, oral mucosal pathology, and the risk of oral squamous cell carcinoma. Inductively coupled plasma mass spectrometry was the predominant analytical platform, while emerging biosensor technologies showed potential for rapid detection and monitoring. Digital and AI-based approaches were identified as promising tools for integrating metallomic data with clinical and molecular biomarkers to support exposure-informed risk stratification. **Conclusions**: Salivary heavy metal profiling represents a biologically informative, non-invasive method for exposure-aware risk assessment in oral health. Although current clinical translation is limited by methodological variability, small cohort sizes, and the lack of standardized reference ranges, integration with digital biosensing platforms and explainable AI frameworks might facilitate scalable, precision-oriented salivary diagnostics.

## 1. Introduction

Saliva is a valuable diagnostic biofluid due to its non-invasive accessibility, repeatable sampling, and ability to reflect local oral processes and systemic biological alterations [1,2,3,4]. While salivary diagnostics has traditionally focused on proteins, nucleic acids, metabolites, and microbiological markers, growing evidence indicates that elemental profiling—particularly the assessment of heavy metals—may provide clinically relevant information regarding oral health status and disease susceptibility [3,4,5].

Heavy metals and related elements, including lead (Pb), cadmium (Cd), mercury (Hg), nickel (Ni), chromium (Cr), arsenic (As), and aluminum (Al), are persistent contaminants with well-documented toxicological effects [5,6,7,8,9]. Exposure may occur through environmental, occupational, dietary, and lifestyle-related pathways. The oral cavity represents a unique interface where systemic exposure converges with local intraoral sources [10,11,12,13]. Dental materials, orthodontic appliances, prosthetic alloys, tobacco products, and e-cigarette aerosols may contribute to continuous low-level metal release directly into the salivary environment [10,11,14].

Saliva integrates these exposures through multiple mechanisms, including passive diffusion from systemic circulation, active secretion by salivary glands, and direct dissolution or corrosion of intraoral metallic components [5,12,13]. Therefore, salivary metal concentrations reflect a compound signal of systemic burden and local release for risk-oriented clinical assessment and biological monitoring [5,13]. Importantly, salivary metals are not biologically inert. Experimental and clinical studies indicate that these metals modulate oxidative stress, inflammatory signaling, immune responses, and oral microbial ecology—pathways central to the development of common oral diseases [14,15,16,17].

Metal-induced oxidative stress represents a key link between exposure and pathology. Redox-active metals promote the generation of reactive oxygen species, impair antioxidant defenses, and induce molecular damage to lipids, proteins, and DNA [15,16,18,19,20]. These processes activate pro-inflammatory signaling cascades and alter epithelial barrier function, contributing to periodontal inflammation, mucosal lesions, and salivary gland dysfunction [16,17]. In parallel, heavy metals act as modifiers for the oral microbiome, selecting for metal-tolerant taxa and reshaping microbial community structure in ways that favor dysbiosis and disease progression [14].

From a diagnostic perspective, these biological effects support the interpretation of salivary heavy metals as biomarkers rather than passive exposure indicators [5]. Altered salivary metal profiles have been associated with dental caries, periodontal disease, allergic and lichenoid mucosal reactions, xerostomia, and markers of genotoxic stress [17,18,21,22,23,24]. Moreover, several metals classified as carcinogenic or potentially carcinogenic have been linked to early molecular events relevant to oral squamous cell carcinoma risk, supporting the use of salivary metal profiling for risk stratification [16,21,22,24].

Advances in analytical chemistry have further strengthened the diagnostic potential of salivary metal analysis. Inductively coupled plasma mass spectrometry (ICP-MS) enables ultra-trace, multi-element detection from small saliva volumes, while emerging biosensor technologies provide a foundation for rapid, chairside screening [3,5,25]. However, clinical translation remains limited by methodological heterogeneity, pre-analytical variability, and the lack of standardized reference ranges [3,5,13].

This comprehensive review evaluates salivary heavy metals as potential diagnostic biomarkers for oral disease. Specifically, it synthesizes evidence regarding analytical detection methods, quantitative concentration ranges, biological mechanisms linking metals to oral pathology, and clinically relevant associations with oral diseases. By enclosing salivary metal profiling within a diagnostic and risk-assessment context, this review aims to clarify its potential role in precision oral diagnostics and personalized dental medicine.

Within this context, salivary heavy metal profiling represents a convergence point between environmental exposure science, oral pathology, and emerging digital diagnostic technologies.

The interpretation of salivary heavy metal profiles is challenged by their multidimensional nature and sensitivity to biological, environmental, and behavioral modifiers, leading to complex, nonlinear relationships that are difficult to resolve with conventional statistical approaches. Metal concentrations in saliva reflect the combined influence of systemic exposure, intraoral sources, inflammatory status, oxidative stress, and microbiome dynamics. In this context, artificial intelligence (AI) and machine learning provide a conceptual framework for integrating heterogeneous salivary data and identifying biologically meaningful patterns relevant to risk stratification. Rather than serving as standalone diagnostic tools, AI-based approaches are best positioned as decision-support systems that contextualize salivary elemental profiles within precision-oriented oral diagnostics.

### 1.1. Saliva as a Biomonitoring Medium

In contrast to blood or urine, which primarily indicate systemic exposure, saliva serves as a composite matrix that reflects both systemic circulation and local release mechanisms [25]. Metals may enter saliva via three principal pathways: passive diffusion of free ionic species from plasma, active secretion by acinar or ductal cells of the salivary glands, and direct dissolution or corrosion of metallic surfaces within the oral cavity. Salivary proteins such as metallothionein, transferrin, and lactoferrin facilitate natural metal chelation and buffering. However, excessive metal accumulation disrupts these protective systems, resulting in protein conformational changes and oxidative damage [26,27,28,29].

Saliva collection is a simple, non-invasive, and repeatable process, making it useful for longitudinal studies, pediatric populations, and occupational surveillance [30]. The concentrations of trace metals in saliva are generally lower than in blood, requiring highly sensitive analytical techniques like inductively coupled plasma mass spectrometry (ICP-MS), atomic absorption spectrometry (AAS), or inductively coupled plasma optical emission spectrometry (ICP–OES) [31,32,33].

### 1.2. Pathophysiological Significance in Oral Health

Metal ions in saliva interact with oral tissues, modulate the oral microbiome, and trigger immunological responses. Mercury vapors from dental amalgam fillings oxidize to form inorganic mercury species that bind to protein thiol groups, altering enzymatic activity and disrupting redox homeostasis [34]. Cadmium and lead can substitute for essential metals such as zinc and calcium in enzymatic binding sites, impairing biological processes including mineralization and epithelial turnover [35,36,37]. Nickel and chromium ions released from orthodontic brackets or prosthetic alloys may provoke hypersensitivity reactions, stimulate cytokine release, and cause cytotoxic effects on oral mucosal cells [17,38,39,40].

In addition to direct biochemical toxicity, metals function as ecological stressors that reshape oral microbial communities. Recent metagenomic studies indicate that even low-level environmental metal exposure can alter the oral microbiome. Davis et al. identified sixteen metals in adult saliva samples and found that elevated salivary concentrations of antimony and mercury correlated with increased *Lactobacillus* abundance and decreased *Granulicatella* abundance, suggesting that metals may shift bacterial community composition toward cariogenic or inflammatory phenotypes [41]. Comparable results have been observed in populations exposed to mining pollution, where prolonged exposure reduced microbial diversity and destabilized microbial co-occurrence networks [42].

### 1.3. Oral Exposure Pathways

The sources of heavy metals in saliva are diverse and often overlapping:

- Environmental exposure: air, soil, and water pollution from industrial activities, mining, and waste incineration contribute to systemic accumulation of metals, which can be excreted into saliva through the salivary glands [43].
- Occupational exposure: workers in metallurgy, battery recycling, and electroplating industries exhibit significantly higher salivary levels of Pb, Cd, and Ni compared to controls [44].
- Lifestyle factors: smoking, e-cigarette use, and certain dietary habits (e.g., consumption of rice or fish contaminated with As or Hg) increase oral exposure [45,46,47,48].

In addition, amalgams, orthodontic wires, and metal-based prosthetic restorations serve as continuous intraoral sources of metal ions, especially under acidic or oxidative conditions [49,50].

### 1.4. Biological Implications

The origins of heavy metals detected in saliva are varied and frequently intersect.

Chronic exposure to metals can disrupt antioxidant systems such as glutathione peroxidase, catalase, and superoxide dismutase, alter salivary flow and composition, and promote inflammatory and mutagenic processes in oral epithelial cells [4,51]. Experimental models indicate that aluminum and cadmium induce salivary gland atrophy and fibrosis, which are associated with elevated oxidative stress markers and pro-inflammatory cytokines [52,53]. These changes may increase clinical susceptibility to caries, periodontitis, and mucosal inflammation.

Because saliva reflects both local and systemic physiological processes, elucidating the impact of heavy metals on its composition and diagnostic utility is of growing importance.

### 1.5. Objectives and Scope of the Review

This comprehensive review aims to:

- summarize current evidence on salivary concentrations of major heavy metals and aluminum in human populations;
- evaluate analytical techniques used for detection and quantification, with emphasis on diagnostic applicability;
- examine associations between salivary metal levels and oral pathophysiological outcomes, including microbial dysbiosis, oxidative stress, and immune dysfunction, and discuss carcinogenic risk–related signals where relevant;
- highlight methodological limitations and propose future directions toward standardization and clinical translation of salivary metal profiling in oral diagnostics.

By combining analytical, mechanistic, microbial, and clinical evidence, this review supports the development of saliva-based methods for risk assessment and monitoring in precision oral diagnostics. Along with new digital biosensing approaches, the manuscript offers a basis for risk stratification and long-term monitoring. This perspective enables precise interpretation of salivary metals and acknowledges current methodological and clinical limits.

## 2. Methodology and Analytical Approaches

### 2.1. Review Design

The present review follows a comprehensive, structured literature review methodology to systematically identify and synthesize clinical and biological evidence regarding salivary heavy metals in oral diagnostics. A narrative approach was considered appropriate given the considerable variability in study designs, analytical methods, exposure assessment strategies, and reported outcomes, which precluded meaningful quantitative synthesis or formal meta-analysis. This approach was chosen to facilitate diagnostic interpretability and translational relevance within a field marked by significant analytical and clinical heterogeneity.

### 2.2. Literature Search Strategy

A comprehensive literature search was undertaken to identify clinical, translational, and experimental studies evaluating heavy metals in saliva and their diagnostic significance for oral and oral–systemic health.

Electronic searches were conducted in the PubMed/MEDLINE database, covering publications from January 2020 to 20 January 2026.

The search strategy incorporated both free-text keywords and controlled vocabulary (MeSH terms, where applicable), structured using Boolean operators (AND/OR). For example, the PubMed search string included: (“saliva” OR “salivary”) AND (“heavy metals” OR “trace elements” OR “metallomics” OR “metal exposure”) AND (“oral health” OR “periodontal disease” OR “oral cancer” OR “oral microbiome” OR “oral inflammation”) AND (“biomarker*” OR “diagnostic*” OR “risk assessment”).

Additional keywords related to analytical techniques and digital diagnostics were included as needed, including ICP-MS, atomic absorption spectrometry, ICP-OES, biosensors, point-of-care testing, artificial intelligence, and machine learning.

Google Scholar served as a supplementary source to identify additional relevant articles and literature not indexed in conventional biomedical databases.

Only articles published in English were included. Conference abstracts lacking full-text availability were excluded due to insufficient methodological detail.

### 2.3. Study Selection Criteria

The study selection process followed a structured approach to ensure comprehensive identification of clinically and biologically relevant evidence regarding salivary heavy metal profiling in oral diagnostics.

Inclusion criteria: studies were considered eligible if they satisfied all of the following conditions:

1. Study type

- Human clinical, observational, or translational studies reporting quantitative measurements of heavy metals or aluminum in saliva;
- Animal or in vivo experimental studies are included when they provide mechanistic, analytical, or translational support directly relevant to salivary metal diagnostics.

2. Analytical methodology

- Use of validated analytical techniques for metal detection, including inductively coupled plasma mass spectrometry (ICP-MS), atomic absorption spectrometry (AAS), inductively coupled plasma optical emission spectrometry (ICP-OES), or equivalent certified methodologies;
- Studies employing emerging biosensor technologies were included when analytical performance and validation parameters were described.

3. Outcome relevance

- Assessment of oral health–related clinical outcomes (e.g., periodontal disease, dental caries, oral mucosal lesions, oral squamous cell carcinoma);
- Evaluation of biologically relevant endpoints, including oxidative stress markers, inflammatory mediators, microbiome composition, genotoxic or epigenetic indicators relevant to oral pathology.

4. Publication characteristics

- Peer-reviewed articles published in English between January 2020 and January 2026;
- Full-text availability with sufficient methodological detail regarding saliva collection, sample handling, and analytical procedures.

Exclusion criteria

Studies were excluded if they met any of the following criteria:

- In vitro or animal studies without clear translational relevance to human salivary diagnostics;
- Occupational or environmental exposure studies lacking salivary measurements;
- Review articles, editorials, commentaries, or conference abstracts without primary analytical data (used only for contextual discussion);
- Studies with insufficient methodological detail concerning saliva collection protocols, contamination control, or metal quantification methods.

Selection process and study categorization: Titles and abstracts identified through database searches were screened for relevance to the review objectives. Full texts of potentially eligible articles were then evaluated according to the inclusion and exclusion criteria.

As a result of this process, thirteen human clinical studies and six animal or in vivo studies were included in the qualitative synthesis. In vitro studies employing artificial saliva or saliva-simulating models were considered separately to support analytical and mechanistic interpretation; however, these were not incorporated into the primary clinical evidence synthesis.

Due to substantial heterogeneity in study design, exposure context, analytical platforms, and reported outcomes, a narrative synthesis was conducted instead of quantitative pooling.

### 2.4. Data Extraction and Synthesis

Data were extracted qualitatively from eligible studies, focusing on:

- Population characteristics and exposure context;
- Saliva collection protocols and pre-analytical conditions;
- Analytical methods and detection limits;
- Reported salivary metal concentration ranges;
- Associations with oral clinical outcomes, inflammatory markers, microbiome composition, or genotoxic indicators.

Given the heterogeneity in study designs, exposure contexts, and analytical methodologies, a formal meta-analytic risk-of-bias assessment was not begun. Instead, studies were qualitatively evaluated based on sample size, analytical validity, and clarity of saliva collection and processing protocols.

Owing to substantial methodological variability across the included studies, findings were synthesized narratively to identify consistent diagnostic patterns, mechanistic relationships, and clinically relevant trends, rather than being pooled quantitatively.

### 2.5. Ethical Considerations

As this review analyzed data exclusively from previously published studies, no new ethical approval for human or animal subjects was required. All information was obtained from the peer-reviewed scientific literature accessed through institutional subscriptions or open-access sources.

Salivary heavy metal assessment offers value beyond serving as a basic exposure indicator. Saliva, which is in constant contact with oral tissues and acts as an interface between systemic circulation and the local mucosal environment, enables the measurement of metal concentrations that reflect ongoing pathophysiological processes. Salivary biomarkers, including malondialdehyde (MDA), pro-inflammatory cytokines, micronucleus frequency, and oxidative DNA damage markers such as 8-OHdG, may function as indicators of metal-induced toxicity. Therefore, saliva can capture external exposure and early molecular changes that occur before the onset of clinically apparent oral disease, underscoring its potential as a non-invasive diagnostic matrix for environmental and oral health monitoring.

## 3. Analytical Techniques for Detecting Metals in Saliva

### 3.1. Inductively Coupled Plasma Mass Spectrometry (ICP-MS)

Inductively coupled plasma mass spectrometry (ICP-MS) is widely regarded as the gold standard for trace metal detection in saliva. Its principal advantages include ultra-low detection limits (parts per trillion), multi-element capability, and isotopic discrimination [54,55,56,57]. Previous studies by Davis et al. and Romano et al. employed ICP-MS to simultaneously quantify more than 20 elements in microliter volumes of saliva, showing its suitability for complete salivary elemental profiling [31,41]. The high sensitivity of ICP-MS enables the detection of subtle and transient variations in metal concentrations associated with dietary intake, smoking, or dental procedures.

Despite its analytical exactness, ICP-MS requires costly instrumentation, specialized technical expertise, and stringent contamination control. Variability during sample preparation procedures—including filtration, centrifugation, and acid dilution—is a major contributor to inter-study heterogeneity and limits direct comparison of quantitative results across investigations [58,59,60].

### 3.2. Atomic Absorption Spectrometry (AAS)

Before the widespread adoption of ICP-MS, atomic absorption spectrometry (AAS) was the most commonly used technique for determining mercury, lead, and cadmium in biological fluids. Cold-vapor AAS (CV-AAS) has been widely used to quantify mercury released from dental amalgam restorations [61]. Flame AAS (F-AAS) is suitable for elements present at higher concentrations, such as copper, iron, and zinc, but lacks the sensitivity required for ultra-trace metal detection.

Although AAS remains a robust and cost-effective analytical approach, its principal limitation is its single-element detection capability, which limits its utility for comprehensive diagnostic profiling. Furthermore, the complex protein-rich matrix of saliva often necessitates chemical modification or extraction steps to reduce spectral and matrix interferences [62].

### 3.3. Inductively Coupled Plasma Optical Emission Spectrometry (ICP-OES)

Inductively coupled plasma optical emission spectrometry (ICP-OES) enables simultaneous multi-element analysis based on characteristic optical emission spectra. Compared with ICP-MS, ICP-OES offers higher detection limits but provides adequate sensitivity for elements at moderate concentrations. This technique has been applied in studies evaluating salivary calcium, magnesium, and iron levels in relation to periodontal and other oral pathologies [63,64,65].

### 3.4. Speciation Analysis

The biological behavior and toxicity of metals depend strongly on their chemical speciation. For arsenic and mercury, species such as As(III), As(V), and methylmercury exhibit distinct toxicokinetic and biological profiles. High-performance liquid chromatography coupled with ICP-MS (HPLC–ICP-MS) enables separation and quantification of individual metal species in biological matrices, including saliva [66,67]. Wang et al. identified both inorganic and methylated arsenic species in human saliva, suggesting that saliva may serve as a dynamic medium reflecting arsenic metabolism and biotransformation [68].

### 3.5. Biosensors and Emerging Technologies

Recent developments in biosensor technology have provided promising alternatives for rapid, in situ detection of heavy metals in saliva. Electrochemical, optical, and colorimetric biosensors utilizing nanomaterials such as graphene derivatives, gold nanoparticles, and quantum dots have demonstrated enhanced sensitivity, portability, and cost efficiency, positioning them as powerful candidates for point-of-care and chairside applications [69,70,71,72,73]. Multiple experimental platforms have achieved detection limits below one part per billion for metals such as lead and mercury; however, large-scale clinical validation remains limited [74].

In addition to analytical performance, the translational relevance of salivary metal biosensors now relies on their ability to integrate with digital systems. Modern biosensing platforms frequently incorporate automated signal acquisition, wireless data transmission, and compatibility with cloud-based storage systems [75]. These features support longitudinal monitoring and standardized data capture across clinical and research environments, thereby addressing persistent challenges related to data fragmentation and inconsistent reporting.

Structured digital data integration is essential for enhancing the interpretability and clinical utility of salivary metal measurements. Standardized recording of key variables, such as the specific metal analyzed, analytical method, saliva collection protocol, sample handling conditions, and clinical context, would improve reproducibility and facilitate meaningful comparisons across studies. Proposed conceptual frameworks for standardized data structuring aim to align analytical results with clinical interpretation, especially in complex exposure scenarios involving multiple metals and biological modifiers.

When integrated with digital acquisition systems, biosensor-derived salivary metal data can serve as a scalable foundation for computational analyses, including trend detection and multivariable risk assessment. Currently, these approaches should be regarded as complementary to, rather than replacements for, established spectrometric methods such as ICP-MS. Although biosensors provide advantages in speed and accessibility, their main utility at present is in screening, repeated monitoring, and incorporation into digitally enabled diagnostic workflows, rather than in definitive quantitative assessment [76].

In summary, biosensors and digital integration strategies constitute a critical translational link between analytical chemistry and precision oral diagnostics. Successful clinical adoption will require further methodological standardization, rigorous validation against reference techniques, and the creation of interoperable data structures that support longitudinal and multidimensional interpretation.

Given the heterogeneity of analytical platforms used in salivary metallomics research, direct comparison of reported concentration ranges across studies requires careful methodological consideration. Key analytical characteristics of commonly employed detection technologies are summarized in Table 1.

Differences in analytical sensitivity, matrix interference profiles, and calibration strategies substantially contribute to inter-study variability in reported salivary metal concentrations and complicate the establishment of universally applicable reference ranges.

### 3.6. Pre-Analytical Variables and Standardization Challenges

Saliva is a dynamic and heterogeneous biofluid, with its composition affected by various physiological and environmental factors such as flow rate, circadian rhythm, hydration status, dietary intake, oral hygiene, and inflammation [77]. The lack of standardized protocols for saliva collection and processing results in considerable analytical variability.

Beyond basic collection variables, analytical standardization presents additional challenges in salivary heavy metal research. Saliva is a protein-rich matrix that may generate matrix effects, ion suppression, or spectral interferences depending on the analytical platform used. In inductively coupled plasma mass spectrometry (ICP-MS), polyatomic interferences and matrix-dependent signal suppression may affect trace-level quantification if appropriate internal standards and matrix-matched calibration are not applied. Atomic absorption spectrometry (AAS), particularly graphite furnace AAS, may be influenced by background absorption and requires careful optimization of temperature programs and chemical modifiers.

Emerging biosensor platforms introduce additional variability related to surface functionalization stability, signal drift, and cross-reactivity with other ionic species present in saliva. Calibration strategies, blank controls, and certified reference materials remain essential to ensure analytical accuracy and cross-study comparability across different detection technologies.

Major pre-analytical factors include:

- Type of saliva: Stimulated and unstimulated samples differ in both dilution and ionic composition.
- Collection devices: certain polymers may adsorb or leach trace metals; the use of acid-washed polypropylene tubes reduces contamination risk;
- Timing of collection: Samples collected after eating or smoking may show temporarily increased metal concentrations.
- Storage conditions: Immediate freezing at −20 °C or below is recommended to prevent precipitation or oxidation of metal species.

Given the substantial influence of pre-analytical variability on salivary metal quantification, structured methodological guidance is essential to improve reproducibility and cross-study comparability [61,62]. Key best-practice considerations are summarized in Box 1.

Box 1 **Recommended Best-Practice Considerations for Salivary Heavy Metal Analysis.**

The following methodological recommendations aim to improve analytical reliability, minimize pre-analytical variability, and strengthen cross-study comparability in salivary heavy metal research:

- Use unstimulated whole saliva for baseline exposure assessment, since stimulated saliva can introduce dilutional variability.
- Collect samples in a fasting state and avoid food intake, smoking, or oral hygiene procedures for at least 1–2 h prior to sampling.
- Standardize collection timing, such as morning sampling, to reduce circadian variability in salivary flow rate and composition.
- Use certified, acid-washed polypropylene tubes to minimize contamination and adsorption of trace metals to collection materials.
- Avoid cotton-based or polymeric collection devices, as these materials may leach metal ions or bind trace elements.
- Process samples immediately or freeze at −20 °C or lower to prevent oxidative transformation or precipitation of metal species.
- Record essential metadata such as dietary intake, smoking status, occupational exposure, dental restorations, recent dental procedures, medication use, and salivary flow characteristics.
- Implement analytical quality control measures, including blank controls, calibration standards, matrix-matched calibration when feasible, and certified reference materials when available.
- Clearly report saliva type (stimulated vs. unstimulated), collection protocol, storage duration, and analytical platform to facilitate reproducibility and enable meta-analytic synthesis.

## 4. Quantitative Ranges of Metals in Saliva

Reported salivary concentrations of heavy metals vary considerably across studies, reflecting differences in analytical methodologies, population characteristics, exposure sources, and pre-analytical conditions. Rather than representing fixed diagnostic thresholds, the values reported in the literature provide approximate concentration ranges that may support comparative interpretation and exposure assessment. Typical salivary concentrations observed in adults without known occupational or environmental exposure are summarized in Table 2.

These concentration ranges should be interpreted carefully, as analytical sensitivity, saliva stimulation status, sampling protocols, and environmental background can substantially influence measured values. Importantly, these ranges do not represent diagnostic reference intervals, but illustrate the variability reported across human studies.

Observational human studies have typically identified associations between salivary metal concentrations and biological alterations within approximate concentration intervals rather than defined thresholds. For instance, elevated salivary oxidative stress biomarkers, including malondialdehyde (MDA) and 8-hydroxy-2′-deoxyguanosine (8-OHdG), have been frequently documented in cohorts with salivary cadmium concentrations of 2–3 µg/L or higher, lead concentrations of approximately 8–10 µg/L, and mercury concentrations exceeding 5 µg/L, particularly among environmentally or occupationally exposed populations. In addition, transient increases in nickel concentrations of 20–25 µg/L have been observed following orthodontic appliance placement, which were associated with short-term inflammatory responses.

The establishment of standardized reference ranges stratified by age, sex, physiological status, and geographic background represents a critical prerequisite for the clinical application of salivary metal analysis. Harmonization of analytical methods and reporting units will be essential to advance saliva from an exposure-monitoring matrix to a reliable diagnostic tool for metal-related oral risk assessment.

### 4.1. Temporal Interpretation of Salivary Metal Concentrations

Interpretation of salivary metal concentrations requires a defined temporal framework, as saliva reflects exposure dynamics differently than blood or urine.

For clarity, exposure is stratified into three temporal categories: transient (hours to days), intermediate (days to weeks), and chronic (months to years). Transient exposure typically results from recent dietary intake, smoking, or short-term environmental contact, leading to temporary fluctuations in salivary metal concentrations. Intermediate exposure involves repeated or sustained contact over days or weeks and may be observed as moderately elevated or variable salivary levels. Chronic exposure, defined as repeated or sustained exposure over months to years and potentially associated with cumulative biological effects, may alter salivary metal profiles.

In contrast to blood, which primarily reflects recent systemic absorption, and urine, which indicates excretory dynamics and systemic elimination, saliva serves as a composite matrix that integrates recent systemic diffusion, active glandular secretion, and local intraoral release. Consequently, salivary metal concentrations likely reflect short-term fluctuations superimposed on longer-term exposure patterns, rather than providing a direct measure of cumulative body burden.

This temporal distinction is especially important for longitudinal monitoring and risk stratification. Repeated salivary measurements can reveal exposure trends and biological response trajectories, while single measurements require cautious interpretation due to potential influence from recent behavioral and environmental factors.

### 4.2. Need for Population-Specific Reference Ranges

Salivary metal concentrations exhibit considerable variability across populations, influenced by factors such as geographic location, environmental pollution, occupational exposure, dietary habits, socioeconomic status, and access to dental care. Therefore, concentration ranges established in one region or cohort should not be directly applied to other populations without appropriate analysis.

Environmental background exposure varies substantially between urban and rural environments, industrial and non-industrial areas, and regions with differing water quality or soil contamination. Occupational groups involved in mining, manufacturing, welding, or battery production often display higher baseline salivary metal concentrations compared to the general population. Additionally, dietary patterns such as seafood consumption, use of metal-containing utensils, and overall nutritional status can affect salivary metal levels.

Age and sex further contribute to variability in salivary metal concentrations, as salivary flow rate, hormonal regulation, and glandular function change throughout the lifespan. As a result, pediatric, geriatric, and medically compromised populations may require separate reference intervals.

Developing population-specific reference ranges that account for demographic and environmental variables is essential for the clinical application of salivary metallomics. Large-scale epidemiological studies employing standardized sampling and analytical protocols are required to establish reliable baseline intervals and exposure-informed risk thresholds.

## 5. Biological Mechanisms Underlying Metal-Induced Oral Effects

Disruption of redox balance is a primary mechanism underlying heavy metal toxicity in the oral cavity. Redox-active metals such as cadmium (Cd), chromium (Cr), nickel (Ni), lead (Pb), and mercury (Hg) increase the production of reactive oxygen species (ROS) via Fenton-like reactions, mitochondrial dysfunction, and depletion of intracellular antioxidant defenses. The resulting accumulation of hydroxyl radicals (•OH) and superoxide anions (O_2_•^−^) promotes lipid peroxidation, protein oxidation, and DNA damage, thereby impairing cellular homeostasis [9,78].

Cadmium toxicity primarily occurs through indirect oxidative mechanisms, including depletion of glutathione and inhibition of antioxidant enzymes such as catalase and superoxide dismutase [79]. Hexavalent chromium (Cr(VI)) is reduced intracellularly to Cr(III), a process that generates ROS and induces DNA–protein cross-links [80,81]. Lead exacerbates oxidative stress by inhibiting δ-aminolevulinic acid dehydratase, which increases free-radical production. Mercury binds to sulfhydryl groups in mitochondrial membranes, disrupting oxidative phosphorylation and further amplifying ROS generation [7,82,83].

Elevated salivary concentrations of oxidative stress biomarkers, including malondialdehyde (MDA) and nitric oxide, have been observed in individuals with increased salivary heavy metal exposure. The presence of these biomarkers suggests that systemic oxidative stress is reflected locally within the oral cavity, thereby strengthening the diagnostic value of salivary analysis [84,85].

Table 3 summarizes the biological mechanisms underlying metal-induced oral toxicity.

In summary, heavy metals contribute to oral toxicity through interconnected mechanisms involving oxidative stress, inflammation, mitochondrial dysfunction, and genomic damage. These overlapping pathways support each other, creating a self-perpetuating inflammatory and dysbiotic environment in the oral cavity [86,87]. The detection of oxidative biomarkers in saliva emphasizes the potential of salivary metal measurements as indicators of environmental exposure and as biologically meaningful signals of early oral and systemic pathology [84,85]. Ongoing research into these mechanisms will further establish saliva as a non-invasive diagnostic tool for monitoring metal-related oral health.

To improve structural clarity and facilitate comparison between shared and metal-specific biological effects, a metal-centered synthesis of dominant mechanisms and salivary correlates is presented in Table 4.

The mechanisms summarized above frequently overlap and interact. These associations are derived primarily from experimental and observational studies and should be interpreted within the context of heterogeneous exposure levels and analytical variability.

## 6. Heavy Metals and Oral Microbiome Dysbiosis: Ecological and Functional Implications

### 6.1. Overview of Metal–Microbiome Interactions

The oral microbiome, which comprises more than 700 bacterial species, maintains ecological homeostasis through nutrient competition, host–microbe signaling, and immune modulation [98,99,100]. This equilibrium is highly sensitive to environmental stressors such as heavy metals. Even at subtoxic concentrations, heavy metals can alter microbial diversity and metabolic activity, resulting in ecological instability commonly referred to as dysbiosis [101].

Heavy metals impose selective pressure on microbial communities by favoring taxa that possess metal resistance mechanisms, such as efflux pumps (including *czc*, *cop*, *ars*, and *mer* operons) or metal-binding proteins such as metallothioneins [102,103]. These adaptive strategies facilitate microbial survival under metal stress and are often linked to increased virulence traits or antibiotic resistance [104]. For instance, *Streptococcus mutans* and *Lactobacillus* species, which are primary contributors to dental caries, can tolerate elevated concentrations of zinc and copper and utilize metal-dependent enzymes to maintain carbohydrate metabolism [105,106]. In contrast, commensal genera such as *Granulicatella* and *Rothia* are more sensitive to metals and tend to decline as salivary metal concentrations rise [103].

Shifts in microbial composition driven by heavy metals disrupt ecological balance and promote dysbiotic states that are associated with dental caries, periodontal inflammation, and oral mucosal pathology [107,108].

### 6.2. Ecological Evidence from Human Studies

Chronic low-level exposure to heavy metals can result in a distinct microbial adaptation state known as the oral metallobiome, which is characterized by microbial communities that persist under sustained ionic stress [109,110,111]. This adaptation tends to favor opportunistic and pro-inflammatory taxa, such as *Fusobacterium*, *Prevotella*, and *Veillonella*, thereby increasing the inflammatory potential within the oral ecosystem [112].

Davis et al. (2020) conducted the first high-resolution analysis of the relationship between salivary metal content and oral microbiome composition [41]. Using inductively coupled plasma mass spectrometry (ICP-MS), they identified multiple metals in adult saliva and demonstrated that higher concentrations of antimony and mercury correlated with reduced microbial diversity and altered community structure. Similarly, Pei et al. (2023) observed decreased alpha diversity and disrupted microbial network topology in individuals with chronic exposure to mining-related metal pollution [42]. Network analyses indicated altered co-occurrence patterns, reflecting reduced ecological resilience and impaired microbial homeostasis [109].

These findings support the concept that salivary metal exposure reshapes oral microbial ecology, diminishing community stability and promoting a pro-inflammatory microenvironment with diagnostic relevance.

### 6.3. Functional and Metabolic Consequences

Heavy metals affect microbial metabolic networks through multiple convergent mechanisms. Toxic metals can substitute for essential cofactors such as iron, manganese, copper, or zinc in microbial enzymes, resulting in impaired enzymatic activity and metabolic inefficiency [113]. Redox-active metals further induce intracellular oxidative stress in microbial cells, thereby modifying redox-sensitive signaling pathways and interspecies interactions [114,115].

Additionally, metals such as nickel and chromium can disrupt quorum-sensing systems, which modulate biofilm formation, microbial communication, and pathogenic potential [116]. Exposure to lead, mercury, or copper has been shown to induce multidrug efflux systems that expel both metals and antibiotics, thereby establishing a mechanistic link between environmental metal exposure and antimicrobial cross-resistance [117,118].

These functional adaptations highlight the long-term ecological impact of heavy metals on the oral microbiome. They may persist even after external exposure has ceased, reinforcing the relevance of microbial signatures as indirect diagnostic indicators of metal-related oral risk [42,119].

## 7. Immune Dysfunction and Inflammatory Pathways

Building upon the general inflammatory mechanisms outlined in Section 5, heavy metals exert more specific immunomodulatory effects within the oral cavity by altering both innate and adaptive immune responses. These effects contribute to persistent mucosal inflammation, impaired immune regulation, and enhanced disease susceptibility.

### 7.1. Immune Activation by Metals

Heavy metals initiate innate immune signaling via pattern-recognition receptors, especially Toll-like receptor 4 (TLR4), which is represented on oral epithelial and resident immune cells. This process induces NF-κB–dependent transcription of pro-inflammatory cytokines, such as IL-1β, IL-6, and TNF-α [120]. Nickel functions as a classical hapten, forming metal–protein complexes that elicit delayed-type hypersensitivity reactions, thereby accounting for allergic stomatitis and oral lichenoid lesions linked to metallic dental materials [121]. Mercury and cadmium drive macrophage polarization toward a pro-inflammatory M1 phenotype, increasing nitric oxide production and tissue injury. In contrast, lead and arsenic inhibit regulatory T-cell activity, impairing immune resolution and promoting chronic inflammation [122].

### 7.2. Salivary Immunoglobulins and Innate Defense

Salivary secretory immunoglobulin A (s-IgA) exhibits time-dependent, biphasic dynamics that should be considered when evaluating metal-associated changes. Acute stress responses or short-term environmental stimuli can transiently elevate s-IgA secretion as part of an adaptive mucosal immune response [122]. In contrast, chronic inflammatory conditions, prolonged toxic exposure, or persistent salivary gland dysfunction are generally linked to decreased s-IgA levels, resulting from immune exhaustion, impaired glandular secretion, or dysregulated mucosal immunity. Thus, the direction of s-IgA alteration is determined by both the duration and intensity of exposure.

Within the context of heavy metal exposure, observed reductions in salivary s-IgA in observational studies most likely indicate chronic immune dysregulation rather than acute adaptive responses. Persistent oxidative stress, disruption of the epithelial barrier, and structural changes in salivary glands may collectively impair immunoglobulin secretion and weaken mucosal defense mechanisms [123]. Consequently, s-IgA alterations should be interpreted in relation to a clearly defined exposure timeline and considered alongside other inflammatory and oxidative biomarkers.

### 7.3. Pro-Inflammatory Oxidative Feedback

Metal-induced oxidative stress sustains inflammatory signaling by persistently activating NF-κB and Nrf2 pathways, establishing a self-reinforcing oxidative–inflammatory feedback loop. Excessive reactive oxygen species generation leads to oxidation of salivary lipids and proteins, reflected by elevated malondialdehyde (MDA) and 8-hydroxy-2′-deoxyguanosine (8-OHdG) levels [124]. This microenvironment promotes matrix metalloproteinase activation, collagen degradation, and progressive tissue damage, hallmarks of chronic periodontal inflammation and salivary gland dysfunction.

## 8. Oxidative Stress, Cellular Injury, and Genotoxicity

Heavy metals increase the risk of oral and systemic diseases through multiple mechanisms, including inflammation, cumulative oxidative injury, mitochondrial dysfunction, and direct genotoxic damage. These processes disrupt redox homeostasis, induce DNA instability, and trigger apoptotic signaling pathways in oral epithelial tissues. Numerous cytogenetic and salivary oxidative biomarkers have been established as quantifiable indicators of metal-induced cellular injury. Table 5 summarizes the principal molecular targets and diagnostic biomarkers associated with oxidative stress and genotoxicity in salivary metallomics.

These findings indicate that salivary heavy metals may contribute to oral carcinogenesis through interconnected epigenetic alterations, activation of oncogenic signaling pathways, disruption of the epithelial barrier, and synergistic interactions with microbial carcinogens. These mechanisms support the concept that salivary metallomics should be viewed primarily as a risk-assessment and stratification tool, particularly when integrated with complementary molecular and microbiome biomarkers. Such multimodal diagnostic approaches may ultimately support digitally enabled saliva-based screening frameworks for early identification of individuals at elevated risk of oral cancer.

## 9. Carcinogenic Potential and Metal-Driven Oral Oncogenesis

Several heavy metals and their compounds are classified as carcinogenic to humans by the International Agency for Research on Cancer (IARC). Arsenic, cadmium, hexavalent chromium, and nickel compounds are classified as Group 1 carcinogens, whereas inorganic lead compounds are classified as probably carcinogenic (Group 2A) [136,137,138,139]. Metallic nickel and methylmercury compounds are considered possibly carcinogenic (Group 2B), while metallic mercury and inorganic mercury compounds are currently classified as not classifiable due to insufficient evidence (Group 3) [136,140].

The mechanisms linking metal exposure to carcinogenesis include oxidative DNA damage, interference with DNA repair pathways, chronic inflammation, and aberrant epigenetic regulation [141].

Although the majority of epidemiological studies have focused on systemic malignancies, emerging evidence implicates metal exposure in oral carcinogenesis. Kumar et al. (2024) reported significantly elevated serum and salivary concentrations of cadmium, chromium, and cobalt in patients with oral squamous cell carcinoma (OSCC) compared with healthy controls, suggesting a potential association between metal burden and oral cancer risk [33]. In another investigation, arsenic exposure was correlated with altered expression of oncogenic microRNAs and reduced p53 activity in oral mucosal cells, supporting a mechanistic link between metal exposure and early oncogenic events [142,143].

A condensed overview of the main epigenetic, molecular, and microbiome-related pathways implicated in metal-induced oral cancer risk is presented in Table 6.

However, the specificity of salivary metal biomarkers remains limited due to the multifactorial nature of metal exposure and the influence of numerous environmental, occupational, and physiological confounders [153]. Consequently, salivary metal analysis should be interpreted within a broader diagnostic framework that integrates clinical findings and complementary biomarker data. Extending these observations to other oral diseases further underscores the systemic impact of metal exposure on oral health beyond carcinogenesis [154].

## 10. Correlations with Specific Oral Diseases

### 10.1. Dental Caries

Metagenomic and epidemiological evidence indicate that elevated salivary antimony (Sb) concentrations are associated with increased caries prevalence, potentially mediated by shifts toward acidogenic and aciduric microbial taxa [41,42]. In contrast, essential trace elements like zinc and iron appear to have protective effects by stabilizing enamel structure and modulating microbial adhesion and metabolism [155].

The relationship between salivary metals and dental caries is bidirectional. Altered metal profiles can influence caries susceptibility by reshaping microbial ecology and enamel chemistry, while established carious lesions may enhance local metal retention by lowering pH and increasing protein binding capacity within the lesion environment [41,156]. These interactions support the interpretation of salivary metals as dynamic indicators of caries-associated ecological imbalance.

### 10.2. Periodontal Disease

Multiple investigations have identified distinct salivary ionic profiles in patients with periodontal disease compared to periodontally healthy controls [31,65]. Elevated concentrations of copper, iron, and manganese are consistently observed in active periodontitis, reflecting increased oxidative metabolism, inflammatory burden, and connective tissue degradation associated with chronic periodontal inflammation [157].

After non-surgical periodontal therapy, these metal concentrations typically decrease toward baseline levels, supporting their potential utility as biomarkers of periodontal inflammatory activity and treatment response [158]. Trace metals may therefore function as pathogenic cofactors that promote oxidative stress and as indicators of disease severity. However, inconsistencies in saliva collection protocols, stimulation methods, analytical sensitivity, and reporting units currently limit inter-study comparability and meta-analytic validation [3].

### 10.3. Mucosal Lesions and Allergic Responses

Hypersensitivity reactions to nickel and chromium often present as erythematous, erosive, or lichenoid oral lesions localized to areas in contact with metallic dental restorations or orthodontic components [159,160,161]. Patch testing and elemental analysis frequently confirm localized accumulation of these metals within affected tissues, supporting a causal association [162].

Mercury-associated oral lichenoid lesions have also been documented, with clinical regression observed following removal of amalgam restorations [163]. These findings support the diagnostic relevance of correlating salivary metal profiles with clinical presentation and patient exposure history when evaluating unexplained mucosal pathology.

### 10.4. Salivary Gland Dysfunction

Exposure to aluminum and cadmium disrupts salivary gland architecture and impairs secretory function, resulting in xerostomia and increased susceptibility to dental caries and mucosal inflammation [52,164,165]. Oxidative damage to acinar cells reduces the production of salivary enzymes and mucins, thereby compromising lubrication, digestion, and antimicrobial defense mechanisms [166]. These functional impairments underscore saliva’s dual role as a diagnostic biofluid and a biological target of metal-induced toxicity, strengthening the clinical relevance of salivary metal analysis in patients with salivary gland dysfunction [167].

To consolidate the evidence discussed for dental caries, periodontal disease, mucosal lesions, and salivary gland dysfunction, representative human studies [11,31,41,44,46,47,48,168,169,170,171,172,173,174] investigating salivary heavy metal levels and their oral and biological correlations are summarized in Table 7. To further strengthen the evidence base for the detection and biological relevance of heavy and trace metals in saliva and saliva-related models from 2000 to the present, animal and in vivo experimental studies [52,53,175,176,177,178] providing mechanistic, analytical, or translational support relevant to salivary metal diagnostics are summarized in Supplementary Table S1. Additionally, in vitro and experimental studies [179,180,181,182,183,184,185] investigating metal release into saliva or saliva-simulating media are presented in Supplementary Table S2. Although in vitro studies using artificial saliva do not replicate the complexity of the human oral environment, they provide valuable insight into the potential for intraoral metal release under controlled physicochemical conditions. Beyond studies demonstrating metal ion release from orthodontic appliances, one investigation reported a decrease in chromium and iron surface contributions accompanied by an increase in oxygen content in used compared to new orthodontic appliance components, indicating surface oxidation and corrosion-related transformations during intraoral exposure [186].

## 11. Digital Innovation and AI-Assisted Salivary Metallomics Diagnostics

Salivary diagnostics are rapidly evolving as a result of advances in digital health technologies, high-throughput molecular profiling, and artificial intelligence (AI)-driven clinical decision support. Historically, research in salivary biomarkers has focused on single-analyte biochemical assays. However, recent approaches increasingly prioritize multimodal diagnostic integration, portable biosensing platforms, and computational modeling frameworks. In this context, salivary heavy metal profiling, also known as salivary metallomics, constitutes a promising yet underdeveloped aspect of digitally enabled precision diagnostics.

Although artificial intelligence (AI) and machine learning (ML) are recognized as powerful tools for interpreting complex salivary biomarker datasets and enhancing diagnostic accuracy, there are currently no well-established salivary metallomics datasets available for training predictive models. As a result, most AI-based applications in this area remain conceptual or exploratory, focusing on pattern recognition and hypothesis generation rather than validated clinical prediction.

To better align digital innovation with biological interpretation, Table 8 reframes salivary metallomics from an application-oriented perspective. Rather than emphasizing specific machine learning algorithms, the table highlights how distinct metallomic patterns may function as exposure-informed risk signals within digitally integrated diagnostic systems.

### 11.1. Toward Smart Salivary Biomonitoring Platforms

Conventional detection of trace metals in saliva primarily utilizes laboratory-based analytical techniques, including inductively coupled plasma mass spectrometry (ICP-MS) and atomic absorption spectroscopy. While these methods offer high sensitivity and accuracy, their accessibility is limited by high costs, infrastructure demands, and the requirement for specialized personnel. Consequently, the implementation of scalable screening programs has been restricted.

Recent digital innovations are addressing these limitations through the development of portable, point-of-care biosensing technologies. Electrochemical sensors, lab-on-a-chip microfluidic platforms, and miniaturized spectrometric devices are increasingly investigated for saliva-based diagnostics. These systems have the potential to enable rapid, non-invasive monitoring of salivary biomarkers in decentralized clinical or community environments. Although most current devices target proteins, metabolites, or inflammatory cytokines, future adaptation for elemental exposure panels may facilitate real-time toxicological saliva screening within preventive oral healthcare.

Integrating metallomic detection into portable digital devices may be especially valuable for populations facing chronic environmental exposure, occupational hazards, or elevated risk of systemic inflammatory diseases, as salivary metals could function as early warning indicators.

### 11.2. Digital Biomarkers and Multimodal Diagnostic Integration

A key trend in contemporary diagnostics is the transition from isolated biomarkers to digitally integrated biomarker ecosystems. Heavy metals detected in saliva should be considered within the broader context of the salivary exposome and inflammatory microenvironment, rather than as independent diagnostic indicators. Their diagnostic utility is likely to increase when combined with additional salivary parameters, such as:

- Microbial dysbiosis signatures;
- Oxidative stress markers;
- Cytokine and immune mediator profiles;
- Epigenetic and transcriptomic indicators;
- Clinical oral and systemic metadata.

Digital biomarker platforms facilitate the integration of heterogeneous data streams into clinically actionable diagnostic models. Within this framework, salivary metallomics can provide quantitative exposure-related features that enhance disease risk prediction, especially for oral potentially malignant disorders, oral squamous cell carcinoma, and chronic inflammatory oral–systemic conditions.

The future diagnostic utility of salivary heavy metals is likely to reside in their role as exposure-informed biomarkers within multi-parameter precision risk models, rather than in disease-specific identification.

It is important to emphasize that these multimodal integration frameworks are currently conceptual and exploratory in the context of salivary metallomics. While biologically plausible, most proposed digital integration models have not yet undergone large-scale clinical validation and should therefore be interpreted as translational perspectives rather than established diagnostic tools.

### 11.3. Artificial Intelligence and Machine Learning for Risk Stratification

Artificial intelligence and machine learning (ML) approaches have become essential for interpreting complex salivary biomarker datasets. In contrast to traditional statistical methods, ML models can identify nonlinear relationships among biomarkers, environmental exposures, and disease phenotypes, making them particularly suitable for high-dimensional salivary diagnostics.

Supervised machine learning approaches such as support vector machines, random forests, and gradient boosting algorithms can be trained using normalized metallomic feature matrices combined with demographic, clinical, and inflammatory metadata. In this context, salivary metal concentrations may serve as quantitative exposure-linked features within multidimensional predictive models rather than as isolated diagnostic variables.

AI-assisted salivary metallomics has the potential to support predictive risk stratification pipelines, including:

- Standardized saliva sampling and elemental quantification;
- Normalization of metallomic signatures across demographic and environmental factors;
- Integration with microbiome, inflammatory, and clinical covariates;
- Supervised ML classification of disease risk categories;
- Explainable AI interpretation using feature attribution tools (e.g., SHAP);
- Generation of personalized diagnostic risk scores.

These approaches may be especially valuable for identifying subclinical high-risk individuals, monitoring exposure-associated disease progression, and supporting preventive interventions before irreversible pathology develops.

Despite its conceptual promise, the application of AI to salivary metal diagnostics is constrained by the lack of large, harmonized datasets. Most current studies are cross-sectional, utilize small cohorts, and exhibit variability in sampling protocols and analytical reference standards. These factors limit the development of generalizable models and delay clinical implementation.

Digitally integrated salivary metallomics has the potential to advance preventive oral healthcare by supporting exposure-informed surveillance and personalized clinical decision-making. Instead of offering definitive disease diagnoses, risk-oriented models can facilitate early identification of high-risk patients, longitudinal monitoring of treatment responses, and targeted interventions, including exposure reduction, enhanced periodontal prevention, or oral cancer screening. These decision-support applications are consistent with the ongoing transition toward precision dentistry and proactive oral–systemic health management.

AI-assisted salivary metallomic modeling should be considered a decision-support strategy that complements established clinical examination, imaging, and molecular diagnostics, rather than replacing them. Prospective validation studies and harmonized datasets are required prior to routine clinical implementation.

Salivary metallomic datasets can be used to generate structured feature sets for modeling, such as absolute concentrations, metal-to-metal ratios, composite exposure indices, longitudinal trajectories, and integrated matrices that combine metallomic, inflammatory, microbiome, and clinical metadata. This feature engineering enhances both model robustness and interpretability.

Standardization of metadata accompanying salivary metallomic measurements is also essential. Variables including age, sex, smoking status, occupational exposure, salivary flow rate, sampling protocol, and analytical platform must be harmonized across datasets to support reproducible model training. The absence of standardized metadata limits interoperability between studies and hinders the development of generalizable AI-based diagnostic tools.

Advancement in AI-assisted salivary metallomics will require interoperable data infrastructures that integrate laboratory-generated elemental profiles with electronic health records and digital dental imaging systems. Federated learning, secure multi-center databases, and standardized reporting frameworks can facilitate collaborative dataset expansion while maintaining data privacy.

Emerging digital diagnostic platforms, such as saliva-on-a-chip microfluidic systems, smartphone-integrated electrochemical sensors, and AI-enhanced spectroscopic devices, demonstrate prototype infrastructures capable of incorporating metallomic panels into broader multimodal salivary diagnostics. While most current applications focus on proteins or metabolites, the technological architecture for elemental integration is conceptually compatible with these platforms.

### 11.4. Challenges for Clinical Translation and Digital Readiness

The clinical translation of salivary metallomics as a digital diagnostic domain requires addressing several key barriers, including:

- Lack of standardized saliva collection protocols (stimulated vs. unstimulated, timing, contamination control);
- Heterogeneity in analytical quantification platforms;
- Absence of validated diagnostic thresholds and population reference ranges;
- Confounding effects of diet, smoking, occupational exposure, and oral microbiome variation;
- Limited longitudinal studies linking salivary metal trajectories to clinical outcomes.

Digital innovation alone is insufficient to address these challenges without coordinated efforts in biomarker harmonization, clinical validation, and achievement of regulatory-grade reproducibility.

Advancement in this field will require multi-center cohort studies, integration of salivary metallomics into broader omics frameworks, and the development of explainable, ethically grounded artificial intelligence models to support real-world diagnostic decision-making.

### 11.5. Future Outlook: Salivary Metallomics in Precision Oral–Systemic Healthcare

The integration of salivary biomarker science with digital diagnostics presents a promising direction for preventive oral–systemic medicine. Heavy metal signatures may function as indicators of toxic exposure and as biologically informative signals reflecting oxidative stress, immune disruption, and carcinogenic risk.

With the maturation of point-of-care technologies and increased clinical validation of AI-driven multimodal modeling, salivary metallomics may contribute to next-generation diagnostic systems that enable scalable screening, personalized monitoring, and exposure-informed risk stratification for both oral and systemic diseases.

Digitally enabled salivary diagnostics represents a promising approach for precision public health strategies, integrating environmental exposures, molecular biomarkers, and clinical phenotypes into actionable diagnostic intelligence.

### 11.6. Current Digital and AI Applications in Salivary Diagnostics

Saliva is increasingly recognized as a valuable diagnostic biofluid because of its non-invasive collection, accessibility, and molecular diversity, which facilitate integration with digital and AI technologies. From 2020 to 2025, significant advancements in biosensing platforms, machine learning analytics, and point-of-care (POC) systems have accelerated the clinical application of salivary diagnostics. These developments are particularly important for early detection and risk assessment in oral carcinogenesis and various systemic diseases.

The growing complexity of salivary biomarker profiles, including microRNAs, cytokines, metabolites, and oxidative stress markers, has driven the adoption of AI algorithms for pattern recognition and predictive modeling. Recent reviews document the increasing application of machine learning and deep learning techniques to enhance diagnostic accuracy, especially for oral squamous cell carcinoma (OSCC), where early detection is particularly difficult [187,188]. AI frameworks are capable of integrating multi-omics salivary signatures with clinical variables, thereby improving individualized risk stratification and clinical decision support.

Microfluidic lab-on-a-chip technologies have progressed rapidly, enabling miniaturized and automated detection of salivary biomarkers at the point of care. These platforms provide high sensitivity, require minimal sample volumes, and are compatible with portable digital readout systems. Recent studies underscore their significance in decentralized monitoring and early screening applications [189,190]. Increasingly, these devices are integrated with AI-supported signal processing to enable real-time interpretation of biomarker data.

Mobile health technologies now play a central role in digital saliva diagnostics, as smartphone-based platforms offer accessible interfaces for microfluidic sensing, optical detection, and cloud-based analytics. A landmark study demonstrated that AI-assisted smartphone and microfluidic systems provide a scalable solution for point-of-care screening, particularly in resource-limited settings [191]. These advancements are consistent with the broader movement toward Internet of Medical Things (IoMT) connectivity in biosensing systems [192].

Vibrational spectroscopy techniques, such as Raman and surface-enhanced Raman spectroscopy (SERS), have attracted interest as label-free methods for detecting biochemical changes in saliva. When paired with AI-driven classification models, these techniques have demonstrated promising accuracy in differentiating malignant from premalignant conditions [193,194]. Recent research further supports integrating salivary Raman profiles with advanced deep learning architectures to enhance cancer discrimination [195,196].

Recent reviews indicate that digitally enhanced saliva biosensors are progressing from experimental prototypes to clinically viable platforms. Wearable and AI-assisted biosensor technologies are under investigation for continuous monitoring of disease-associated biomarkers, supporting more proactive healthcare approaches [197,198]. However, challenges persist in standardization, validation across diverse populations, and regulatory implementation.

Table 9 provides an overview of recent studies focused on AI applications specific to oral cancer in salivary diagnostics.

In summary, the years 2020 to 2025 have seen significant progress in AI-enabled salivary diagnostics, driven by advances in biosensor miniaturization, smartphone integration, deep learning analytics, and connected health infrastructures. Ongoing integration of saliva-based biomarker discovery with robust computational models will be critical for translating these technologies into routine clinical practice, especially for oral cancer risk assessment and early intervention.

## 12. Limitations and Clinical Translation Readiness

Heavy metals exert multiple effects within the oral ecosystem by simultaneously altering microbial balance, immune signaling, biochemical pathways, and genetic regulation. Chronic exposure drives oxidative imbalance and epigenetic remodeling, promoting a persistent pro-inflammatory microenvironment that predisposes individuals to a spectrum of oral pathologies. These effects manifest as reduced microbial diversity, proliferation of metal-tolerant taxa, compromised mucosal integrity, and progressive tissue dysfunction. These interactions reveal a continuum of toxicity linking environmental exposure to clinical outcomes, ranging from subclinical salivary gland impairment to overt inflammatory disease and carcinogenesis.

Recognizing saliva not only as a diagnostic biofluid but also as an active participant in metal-induced oral pathology is essential for advancing preventive, predictive, and personalized dentistry. However, despite growing interest in salivary metal analysis, the current body of evidence remains constrained by substantial methodological heterogeneity, relatively small sample sizes, and inconsistent analytical protocols. Most available studies are cross-sectional, lack longitudinal follow-up, and rarely integrate clinical outcomes with molecular or microbiome data. Confounding factors—including smoking, dietary habits, occupational exposure, systemic disease, medication use, and the presence of metallic dental materials—are frequently under-controlled, limiting the robustness of reported associations.

Pre-analytical variability represents an additional challenge. Differences in saliva stimulation, collection timing, storage conditions, and reporting units impede inter-study comparability and preclude the establishment of standardized reference values. Although inductively coupled plasma mass spectrometry (ICP-MS) represents the analytical gold standard, not all studies employ certified reference materials or inter-laboratory quality controls, further limiting reproducibility.

Future research should prioritize harmonized methodologies, larger and more diverse study populations, and longitudinal designs capable of linking elemental exposure to immunological, microbial, and genomic endpoints. Standardized saliva collection protocols—such as the use of unstimulated morning samples collected in acid-washed polypropylene tubes, immediate freezing at ≤−20 °C, and uniform reporting units (µg/L or µg/min)—are critical to ensure reproducibility and inter-laboratory comparability [204]. Adoption of these standards will facilitate accurate population-level biomonitoring and support the development of validated reference ranges for salivary heavy metals.

Despite growing analytical and biological evidence supporting salivary heavy metal profiling, its translation into routine clinical diagnostics remains at an early stage of development. To contextualize the current state of the field and avoid overinterpretation of emerging technologies, the Diagnostic Readiness Levels (DRLs) framework can be used to structure evaluations of translational maturity.

Diagnostic Readiness Levels describe the progression of a biomarker or diagnostic approach from analytical feasibility through clinical validation and utility. When applied to salivary heavy metal analysis and AI-assisted risk stratification, this framework highlights both current achievements and critical gaps that must be addressed before clinical implementation.

Within this diagnostic-readiness context, artificial intelligence should be viewed not as a mature clinical solution but as a necessary analytical framework for managing the multidimensionality of salivary metal data. The following section outlines emerging AI-assisted approaches that are conceptually aligned with the current readiness level of salivary heavy metal profiling and highlights their role as decision-support tools rather than standalone diagnostic systems.

## 13. Clinical Translation and Future Smart Salivary Diagnostics

Recent advances in digital technologies are progressively reshaping the salivary biomarker research. Improvements in sensor miniaturization, signal acquisition, data connectivity, and computational analytics have expanded the feasibility of saliva-based monitoring beyond single, static measurements toward longitudinal and data-rich assessment frameworks. Within this context, artificial intelligence (AI) and machine learning approaches are increasingly being explored as tools to support the handling of the intrinsic complexity, variability, and multidimensional structure of salivary data.

The most tangible intersection between salivary heavy metal analysis and AI currently occurs at the level of biosensor platforms rather than downstream clinical datasets. Recent developments in saliva-based electrochemical and optical sensors have incorporated digital signal processing, automated data acquisition, and algorithm-ready architectures designed to support future machine learning integration. In these systems, computational methods are applied to improve signal stability, reduce noise, and discriminate patterns from complex sensor outputs, rather than to infer disease states directly. The work by Anchidin-Norocel et al. exemplifies this direction by describing saliva-based sensor platforms for continuous heavy metal monitoring in metabolic disorders, highlighting technological feasibility while stopping short of clinical AI-driven decision-making [71]. Representative studies linking salivary biosensing, digital analytics, and AI-oriented methodologies are summarized in Table 10.

The convergence of salivary biomarker science with digital health innovation is reshaping the future of non-invasive diagnostics for oral and systemic disease. While salivary heavy metals have traditionally been investigated primarily as indicators of toxic exposure, new evidence suggests that metallomic signatures may also serve as biologically informative biomarkers reflecting oxidative burden, immune dysregulation, microbiome disruption, and carcinogenic susceptibility within the oral microenvironment.

The clinical value of salivary metallomics is unlikely to lie in isolated elemental measurements, but rather in its integration into multimodal diagnostic ecosystems. Advances in portable biosensing platforms, lab-on-chip technologies, and point-of-care analytical devices offer new opportunities to decentralize salivary metal monitoring beyond specialized laboratory settings. In parallel, artificial intelligence and machine learning approaches are increasingly capable of integrating metallomic profiles with complementary saliva-derived dimensions—including microbiome features, inflammatory cytokines, oxidative stress markers, and transcriptomic signatures—to generate interpretable risk stratification models.

Future progress in this field will require standardized sampling protocols, harmonized analytical pipelines, and large longitudinal cohorts to establish population reference ranges and clinically meaningful thresholds. Moreover, explainable AI frameworks will be essential to ensure that digitally assisted salivary diagnostics remain transparent, clinically interpretable, and ethically deployable in real-world oral healthcare systems. Collectively, these developments highlight salivary metallomics as a promising component of next-generation digital saliva diagnostics, supporting early-exposure-informed surveillance and precision preventive health strategies.

## 14. Conclusions

Salivary heavy metal profiling constitutes a novel diagnostic approach at the intersection of environmental exposure science, oral–systemic pathology, and digital biomarker development. Elements such as cadmium, chromium, arsenic, mercury, lead, and aluminum increase oral disease risk through mechanisms including oxidative injury, epithelial dysfunction, immune modulation, microbial dysbiosis, and epigenetic changes. Despite current clinical evidence being constrained by methodological variability and small sample sizes, salivary elemental profiles demonstrate significant potential as non-invasive biomarkers for exposure-related risk assessment, especially in oral potentially malignant disorders and oral carcinogenesis.

The future diagnostic utility of salivary metallomics will rely on its integration within digitally enabled precision medicine frameworks, rather than its application as an isolated indicator. Incorporating biosensor platforms, point-of-care salivaomics technologies, and artificial intelligence-driven multimodal modeling could facilitate scalable screening, individualized monitoring, and the early identification of high-risk individuals in both oral and systemic disease contexts. As digital advancements enhance salivary diagnostic capabilities, salivary metallomics is positioned to contribute significantly to next-generation diagnostic systems that support preventive oral and systemic healthcare.

## Figures and Tables

**Table 1:** Comparative analytical characteristics of major salivary heavy metal detection platforms.

Analytical Platform	Typical Detection Limit	Multi-Element Capability	Matrix Sensitivity	Calibration Requirements	Advantages	Limitations
ICP-MS	ppt–low ppb	Yes	Moderate (matrix effects possible)	Internal standards; matrix-matched calibration	Extremely high sensitivity; multi-element profiling	Expensive; requires specialized infrastructure
ICP-OES	low ppb	Yes	Moderate	External calibration; possible matrix matching	Simultaneous multi-element detection	Lower sensitivity than ICP-MS
AAS (Flame)	mid–high ppb	No (single element)	Moderate	External calibration; background correction	Cost-effective; widely available	Limited sensitivity; single-element analysis
GFAAS	low ppb	No (single element)	Higher matrix influence	Careful temperature optimization; chemical modifiers	Improved sensitivity vs. flame AAS	Time-consuming; limited throughput
CV-AAS (for Hg)	low ppb	No	Low–moderate	Specific mercury calibration	Highly specific for mercury	Limited to Hg analysis
Electrochemical biosensors	ppb (variable)	Limited (target-specific)	High (saliva composition dependent)	Frequent recalibration; surface validation	Portable; point-of-care potential	Signal drift; cross-reactivity; limited standardization

Abbreviations: ICP-MS, inductively coupled plasma mass spectrometry; ICP-OES, inductively coupled plasma optical emission spectrometry; AAS, atomic absorption spectrometry; GFAAS, graphite furnace atomic absorption spectrometry; CV-AAS, cold vapor atomic absorption spectrometry; ppt, parts per trillion; ppb, parts per billion.

**Table 2:** Typical salivary concentrations in adults without known occupational/environmental exposure.

Metal	Typical Salivary Range (µg/L)	Analytical Method Used	Notes
Lead (Pb)	1–15	ICP-MS, AAS	Higher concentrations reported in smokers and industrial workers [41]
Cadmium (Cd)	<1–5	ICP-MS, GFAAS	Frequently elevated in smokers [42]
Mercury (Hg)	0.5–12	CV-AAS, ICP-MS	Correlates with number of dental amalgam surfaces [44,49]
Nickel (Ni)	2–30	ICP-MS, FAAS	Transient peaks after orthodontic appliance placement [20]
Chromium (Cr)	0.5–10	ICP-MS	Released from dental alloys, particularly under acidic conditions [37]
Arsenic (As)	0.2–4	HPLC–ICP-MS (speciation)	Relative proportions of species vary between individuals [54]
Aluminum (Al)	5–80	ICP-OES, ICP-MS	Elevated levels reported in regions with high aluminum content in drinking water [47]

Abbreviations: ICP-MS, inductively coupled plasma mass spectrometry; AAS, atomic absorption spectrometry; ICP-OES, inductively coupled plasma optical emission spectrometry; CV-AAS, cold vapor atomic absorption spectrometry; GFAAS, graphite furnace atomic absorption spectrometry; HPLC–ICP-MS, high-performance liquid chromatography coupled with ICP-MS.

**Table 3:** Mechanistic pathways and representative salivary correlates.

Mechanism	Key Metals Involved	Biological Effects in Oral Tissues	Supporting Evidence/Biomarkers	References
Oxidative stress generation	Cd, Cr, Ni, Pb, Hg	ROS overproduction, lipid peroxidation, protein oxidation, DNA strand breaks	Increased MDA, nitric oxide in saliva	[9,78,84,85]
Antioxidant depletion	Cd	Glutathione depletion, inhibition of catalase and SOD enzymes	Impaired antioxidant defenses	[79]
Metal-induced DNA damage	Cr(VI)	Intracellular reduction generates ROS, DNA–protein cross-links	Genotoxic instability in oral cells	[80,81]
Enzymatic disruption & ROS amplification	Pb	Interference with δ-aminolevulinic acid dehydratase, enhanced radical formation	Oxidative injury pathways	[7,82]
Mitochondrial toxicity	Hg	Binding to sulfhydryl groups, impaired oxidative phosphorylation	Increased ROS production	[82,83]
Inflammatory and dysbiotic microenvironment	Multiple metals	Chronic inflammation, microbiome disruption, immune dysregulation	Persistent oral inflammatory signaling	[86,87]
Inflammatory pathway activation	Multiple metals	Activation of NF-κB and MAPK pathways; cytokine overexpression	Increased IL-1β, IL-6, TNF-α levels	[88,89]
Salivary gland dysfunction	Al, Cd	Acinar atrophy, fibrosis, reduced salivary flow	Structural gland injury and xerostomia risk	[52,90,91,92]
Impaired xenobiotic clearance	Multiple metals	Reduced metal excretion, prolonged local oral exposure	Increased retention within oral tissues	[51]
Epigenetic dysregulation	As, Cd	Altered DNA methylation and histone acetylation; tumor suppressor silencing	Dysregulated antioxidant and cancer-related genes	[93,94]
Chromosomal/genotoxic injury	Hg	Micronucleus formation in buccal epithelial cells	Cytogenetic marker of genomic instability	[95]
Oxidative DNA damage and carcinogenic progression	Pb	Persistent oxidative DNA lesions, early carcinogenesis, genomic instability	Elevated salivary 8-OHdG levels	[96,97]

Abbreviations: Cd, cadmium; Cr, chromium; Ni, nickel; Pb, lead; Hg, mercury; ROS, reactive oxygen species; MDA, malondialdehyde; SOD, superoxide dismutase; DNA, deoxyribonucleic acid.

**Table 4:** Metal-centered mechanistic synthesis and salivary diagnostic relevance.

Metal	Dominant Biochemical Mechanisms	Primary Molecular Targets	Salivary Diagnostic Correlates
Cadmium (Cd)	Oxidative stress via glutathione depletion; mitochondrial dysfunction; epigenetic modulation	Glutathione system, catalase, mitochondrial membranes, DNA methylation pathways	Elevated MDA, 8-OHdG; reduced antioxidant capacity; potential s-IgA reduction
Lead (Pb)	Enzymatic inhibition; ROS amplification; interference with heme synthesis; genotoxic stress	δ-aminolevulinic acid dehydratase, antioxidant enzymes, nuclear DNA	Increased 8-OHdG; oxidative protein damage; inflammatory cytokine elevation
Mercury (Hg)	Thiol-binding toxicity; mitochondrial impairment; immune dysregulation	Sulfhydryl groups in proteins, mitochondrial membranes, NF-κB pathway	Elevated ROS markers; micronucleus formation; mucosal inflammatory responses
Chromium (Cr VI)	Intracellular reduction generating ROS; DNA–protein cross-links; carcinogenic signaling activation	Nuclear DNA, DNA repair enzymes, PI3K/Akt pathway	Genotoxic endpoints (micronuclei); oxidative DNA damage; pro-inflammatory cytokines
Nickel (Ni)	Hapten formation; TLR4 activation; allergic hypersensitivity; oxidative signaling	TLR4 receptor, NF-κB pathway, epithelial junction proteins	Elevated IL-6, TNF-α; localized mucosal inflammation; transient inflammatory peaks
Arsenic (As)	Epigenetic dysregulation; oxidative stress; oncogenic microRNA modulation	DNA methyltransferases, histone acetylation pathways, p53 signaling	Altered miRNA profiles; oxidative biomarkers; potential carcinogenic risk signals
Aluminum (Al)	Salivary gland toxicity; acinar atrophy; oxidative imbalance	Acinar epithelial cells, antioxidant enzymes	Reduced salivary flow; xerostomia risk; altered salivary protein composition

Abbreviations: ROS, reactive oxygen species; MDA, malondialdehyde; 8-OHdG, 8-hydroxy-2′-deoxyguanosine; NF-κB, nuclear factor kappa-light-chain-enhancer of activated B cells; TLR4, Toll-like receptor 4; PI3K/Akt, phosphoinositide 3-kinase/protein kinase B.

**Table 5:** Oxidative stress, mitochondrial injury, and genotoxic biomarkers associated with salivary heavy metal exposure.

Pathophysiological Target	Key Heavy Metals Involved	Major Molecular Effects	Salivary/Oral Biomarker Indicators	Diagnostic/Clinical Relevance	References
Redox imbalance and ROS generation	Cadmium (Cd), Lead (Pb), Chromium (Cr VI)	Disruption of antioxidant defenses and excessive ROS production; Cd inhibits glutathione synthesis and antioxidant enzymes, while Pb increases free radical burden via δ-aminolevulinic acid dehydratase interference	Elevated oxidative stress markers in saliva (protein carbonyls, 8-OHdG)	Supports oxidative profiling as complementary readout to elemental exposure biomarkers	[111,123,125,126]
Mitochondrial dysfunction and apoptosis	Cd, Pb	Mitochondrial membrane depolarization, reduced ATP synthesis, intrinsic apoptotic activation (caspase-3); oral keratinocytes show DNA fragmentation under metal stress	Apoptotic activation markers (caspase signaling) and oxidative damage indicators	Mitochondrial injury links chronic exposure to tissue degeneration and carcinogenic susceptibility	[127,128]
Mitochondrial DNA vulnerability	Multiple metals	mtDNA is highly susceptible due to proximity to electron transport chain and limited repair capacity; accumulation of mtDNA damage drives senescence and persistent oxidative imbalance	Potential inclusion of mtDNA integrity as future biomarker dimension	Reinforces role of mitochondrial injury in long-term oral tissue dysfunction	[129,130]
Direct genotoxicity and DNA adduct formation	Cr VI, Hg, Cd	Cr VI forms DNA adducts following intracellular reduction to Cr(III); chronic metal exposure promotes strand breaks and chromatin remodeling	Buccal epithelial genotoxic endpoints (micronuclei frequency)	Links salivary metal burden to measurable genomic instability in oral mucosa	[131,132,133]
Cytogenetic monitoring (micronucleus assay)	Hg, Cd	Increased micronucleus frequency in occupationally exposed individuals and those with elevated salivary metals	Buccal micronucleus assay (non-invasive)	Practical diagnostic tool for monitoring cumulative genotoxic exposure	[132,133]
Oxidative DNA and protein damage biomarkers	Multiple metals	Persistent oxidative injury produces lesions such as 8-OHdG and protein carbonyl accumulation, reflecting impaired repair capacity	Salivary 8-OHdG, protein carbonyls	Strengthens diagnostic potential of combined elemental + oxidative biomarker stratification	[134,135]
Early events in malignant transformation	Chronic multi-metal exposure	Progressive oxidative lesions, epigenetic dysregulation, genomic instability constitute early carcinogenic processes	Combined salivary metallomics + oxidative biomarker panels	Supports use of saliva for early oral cancer risk stratification rather than definitive diagnosis	[9,16]

Abbreviations: ROS, reactive oxygen species; Cd, cadmium; Pb, lead; Cr(VI), hexavalent chromium; Hg, mercury; mtDNA, mitochondrial DNA; ATP, adenosine triphosphate; 8-OHdG, 8-hydroxy-2′-deoxyguanosine; SOD, superoxide dismutase; DNA, deoxyribonucleic acid.

**Table 6:** Carcinogenic mechanisms associated with heavy metal exposure.

Carcinogenic Mechanism	Key Heavy Metals Involved	Major Biological Effects	Diagnostic/Clinical Implications	References
Epigenetic dysregulation	Arsenic (As), Cadmium (Cd), Nickel (Ni)	Hypermethylation of tumor suppressor genes (e.g., *p16INK4a*, *RASSF1A*) and hypomethylation of proto-oncogenes; modulation of histone acetylation and microRNA expression sustaining proliferative and anti-apoptotic signaling	Supports use of metallomic profiles in saliva as exposure-linked biomarkers of early carcinogenic risk	[144]
Oncogenic signaling activation	Chromium (Cr VI) and other metals	Activation of PI3K/Akt survival pathway in transformed oral epithelial cells; sustained NF-κB and STAT3 activation through chronic inflammation and ROS production	Metal-driven pathway activation may complement molecular saliva markers for risk stratification	[145,146]
Disruption of epithelial integrity	Multiple metals (As, Cd, Cr)	Impairment of intercellular junctions and increased epithelial permeability, facilitating invasion and malignant progression	Highlights metals as contributors to barrier dysfunction in oral premalignant conditions	[147]
Synergy with microbial carcinogens	Metals promoting dysbiosis (As, Cd, Cr)	Metal-stressed environments favor opportunistic OSCC-associated pathogens such as *Fusobacterium nucleatum* and *Porphyromonas gingivalis*, which secrete endotoxins and proteolytic enzymes promoting DNA damage, inflammatory signaling, and epithelial–mesenchymal transition (EMT)	Emphasizes combined elemental–microbiome diagnostic frameworks for assessing carcinogenic risk	[148,149]
Microbial–chemical carcinogenic axis	Metals amplifying oxidative stress	Oxidative stress enhances microbial carcinogenic effects, suggesting synergistic interactions driving oral cancer initiation and progression	Supports integration of metallomics and microbial signatures within AI-assisted multimodal saliva diagnostics	[150,151]
Clinical biomarker potential	Cd, Cr, As	Salivary metals proposed as non-invasive biomarkers for oral carcinogenic risk assessment rather than definitive disease diagnosis; enhanced sensitivity when combined with salivary microRNA, oxidative stress markers, or inflammatory cytokines	Positions salivary elemental profiling as a tool for early risk stratification in precision oral oncology	[152]

Abbreviations: As, arsenic; Cd, cadmium; Cr VI, hexavalent chromium; Ni, nickel; PI3K, phosphoinositide 3-kinase; Akt, protein kinase B; NF-κB, nuclear factor kappa-light-chain-enhancer of activated B cells; STAT3, signal transducer and activator of transcription 3; ROS, reactive oxygen species; OSCC, oral squamous cell carcinoma; EMT, epithelial–mesenchymal transition; miRNA, microRNA; AI, artificial intelligence.

**Table 7:** Representative studies assessing heavy metals in human saliva and associated oral and biological outcomes (2000–present).

Study/Year	Population/Sample Size	Analytical Method	Metals Assessed	Main Findings	Associated Oral/Biological Outcomes
Pizzichini et al., 2000 (Italy) [168]	34 healthy adults with amalgam fillings	Vapor AAS	Hg	Salivary Hg correlated with surface area of amalgam; in females, negative correlation between salivary antioxidant activity and Hg/amalgam metrics.	Links Hg in saliva to a biological effect marker (oxidative stress), not just concentration.
Leistevuo et al., 2001 (Finland) [169]	88 adults with amalgam fillings vs. control	CV–AAS	Organic and inorganic Hg	Salivary Hg positively correlated with number of amalgam-filled tooth surfaces.	Amalgam restorations identified as continuous intraoral organic Hg source.
Costa de Almeida et al., 2009 (Brazil) [170]	144 children	ICP-MS	Pb	Pb levels in saliva reflected environmental exposure.	Salivary Pb correlated with ambient contamination but not with enamel Pb.
Kim et al., 2010 (Korea) [46]	30 smokers vs. non-smokers	ICP-MS	Trace metals	Smokers showed higher salivary cotinine and Al concentrations.	Dose-dependent relationship between smoking intensity and salivary metal burden.
Gil et al., 2011 (Spain) [171]	178 workers	AAS	Cd, Cr, Mn, Ni, and Pb	Mean blood and urine levels within biological exposure indices.	Longer occupational exposure associated with higher Mn and Ni in saliva.
Dwivedi et al., 2015 (India) [172]	13 orthodontic patients	AAS	Ni, Cr	Salivary Ni and Cr peaked at one-week post-appliance placement and then declined.	Levels below toxicity thresholds; relevant in Ni-sensitive individuals.
Junaid et al., 2016 (Pakistan) [44]	75 metal industry workers vs. control	ICP-MS	Heavy metals	Significantly higher salivary metal concentrations in exposed workers.	Associated with elevated oxidative stress biomarkers (MDA, protein carbonyls).
Wang et al., 2017 (China) [68]	70 adults	HPLC–ICP-MS	As(III), As(V), methylated As	Salivary As strongly correlated with urinary and water As concentrations.	Evidence of dynamic As metabolism and excretion in saliva.
Quadras et al., 2019 (India) [11]	50 orthodontic patient vs. control	AAS	Ni, Cr, Zn	Transient increase in salivary Ni, Cr, Zn after appliance placement.	Values below dietary intake; no systemic toxicity observed.
Davis et al., 2020 (USA) [41]	61 children and adults	ICP-MS	Heavy metals	Distinct bacterial taxa associated with Sb, As, and Hg levels.	Sb linked with higher caries prevalence and microbial dysbiosis.
Romano et al., 2020 (Italy) [31]	46 periodontitis patient vs. control	ICP-MS	Li, Na, Mg, Mn, Fe, Cu, Zn, Ca, K, Rb	Altered ionic profile observed in periodontitis.	Cu, Fe, and Mn correlated with inflammation and periodontal severity.
Tomova et al., 2024 (Bulgaria) [173]	18 subjects with metallic crowns vs. control	ICP-MS	Cr, Co	Elevated Cr in unstimulated saliva of patients with metal restorations.	Pre-treatment screening recommended to minimize allergic or cytotoxic effects.
He et al., 2025 (USA) [47]	38 e-cigarette user vs. 18 control	ICP-MS	Trace metals	Significantly elevated salivary metals in heavy e-cigarette users.	Correlated with pro-inflammatory cytokines and oxidative markers (MDA, 8-OHdG).
Tobia et al., 2025 (Italy) [174]	46 Pb exposed workers vs. control	ICP-MS	Pb	Mean salivary Pb levels were significantly higher in exposed workers	A moderate correlation was found between environmental and salivary lead levels

Abbreviations: CV-AAS, cold vapor atomic absorption spectrometry; ICP-MS, inductively coupled plasma mass spectrometry; AAS, atomic absorption spectrometry; HPLC–ICP-MS, high-performance liquid chromatography coupled with ICP-MS; MDA, malondialdehyde; 8-OHdG 8-hydroxy-2′-deoxyguanosine.

**Table 8:** Application-oriented digital integration of salivary metallomic patterns.

Salivary Metallomic Pattern	Associated Biological Domain	Digital Interpretation Role	Potential Clinical Application
Multi-metal oxidative stress signature (elevated Cd, Pb, Hg)	Oxidative DNA damage, mitochondrial dysfunction	Feature aggregation into composite oxidative-metal indices	Oral cancer risk stratification; monitoring inflammatory burden
Transient Ni or Cr elevation following orthodontic exposure	Acute inflammatory signaling	Short-term exposure detection and temporal trend modeling	Monitoring orthodontic biocompatibility
Chronic Cd/Pb elevation with reduced s-IgA	Immune dysregulation	Longitudinal immune-exposure correlation analysis	Periodontal disease progression monitoring
Metal-associated microbiome shifts (e.g., Sb, As, Hg correlations)	Dysbiosis and ecological instability	Multimodal clustering (metallomics + microbiome)	Caries and mucosal pathology risk prediction
Elevated As with epigenetic biomarkers	Epigenetic modulation and oncogenic signaling	Integration into multi-parameter carcinogenic risk models	Early OSCC susceptibility screening
Multi-metal low-level chronic exposure pattern	Subclinical inflammatory microenvironment	Population-level exposure-informed screening dashboards	Preventive precision dentistry

Abbreviations: Cd, cadmium; Pb, lead; Hg, mercury; Ni, nickel; Cr, chromium; As, arsenic; Sb, antimony; s-IgA, secretory immunoglobulin A; OSCC, oral squamous cell carcinoma.

**Table 9:** Oral Cancer–Specific AI Applications in Salivary Diagnostics (2020–2025).

Study	Salivary Target/Sample Type	Digital/AI Approach	Key Findings/Clinical Relevance
Borșa et al. (2023) [199]	Filtered saliva metabolites (oral cancer vs. controls)	SERS biochemical profiling + computational spectral interpretation	Identified discriminatory salivary Raman bands linked to oral cancer metabolites, supporting AI-driven biomarker discovery.
Faur et al. (2023) [200]	Salivary exosomes (oral & oropharyngeal SCC)	Surface-enhanced Raman spectroscopy (SERS) + chemometric multivariate classification (PCA-LDA)	Successfully discriminated SCC patients from healthy controls using AI-supported spectral profiling of exosomes.
Hanna et al. (2024) [194]	Saliva-based Raman liquid biopsy (OSCC & OPMDs)	Deep learning–enhanced vibrational spectroscopy (reviewed applications)	Reported strong potential of AI-Raman approaches for early oral cancer detection and monitoring progression.
Liu et al. (2024) [201]	Salivary exosome SERS (multiple cancers incl. OSCC)	Machine learning fingerprint analysis for exosomal Raman features	Emphasized AI-supported SERS as a next-generation liquid biopsy approach for non-invasive cancer diagnostics.
Kaushik et al. (2025) [196]	Whole saliva (healthy vs. premalignant vs. malignant OSCC)	Portable SERS platform + multivariate machine learning analysis	Demonstrated accurate distinction of premalignant and malignant stages, supporting point-of-care oral cancer screening.
Wang et al. (2026) [202]	Salivary microbiome signatures (cross-cohort OSCC datasets)	Machine learning classifiers trained on microbial composition patterns	Developed a robust non-invasive diagnostic model across cohorts (*n* = 665), highlighting microbiome-based AI screening potential.
Zhao et al. (2026) [203]	Salivary exosome phenotyping	Machine learning–assisted exosome classification platforms	Highlighted ML-enabled exosome profiling as an advanced strategy for cancer screening and immune-marker detection.

Abbreviations: AI, Artificial Intelligence; ML, Machine Learning; SERS, Surface-Enhanced Raman Spectroscopy; PCA-LDA, Principal Component Analysis–Linear Discriminant Analysis; OSCC, Oral Squamous Cell Carcinoma; OPMDs, Oral Potentially Malignant Disorders.

**Table 10:** Representative studies applying artificial intelligence, machine learning, or digital analytics to salivary biomarker data.

Study	Saliva Data Type	Analytical Method Used AI/ML or Digital Component	Main Application
Kumar et al. 2024 [1]	Salivary biomarker panels	AI predictive models	Early detection of systemic disease
Adeoye and Su 2024 [205]	Multi-omic salivary biomarkers (proteins, nucleic acids, metabolites, microbiome)	Review of ML approaches (SVM, Random Forests, Neural Networks)	Biomarker discovery and validation in oral diseases
Anchidin-Norocel et al. 2024 [71]	Salivary heavy metal sensor outputs	Digital signal processing; AI-ready sensor platforms	Continuous monitoring of heavy metals linked to diabetes
Sun et al. 2025 [206]	Proteins, metabolites, microbiome features	Random Forests, Support Vector Machines, Neural Networks	Disease screening and classification
Dal’Ongaro et al. 2025 [207]	FTIR spectral profiles of saliva	Machine learning classification (unspecified models)	Screening of congenital syphilis
Garcia-Junior et al. 2025 [198]	Electrochemical salivary sensor signals	SVM, Neural Networks	AI-assisted signal interpretation
Sharma et al. 2025 [208]	Saliva sensing + pattern recognition	Machine learning	Real-time saliva health monitoring

Abbreviations: AI, Artificial Intelligence; ML, Machine Learning; SVM, Support Vector Machine; FTIR, Fourier Transform Infrared spectroscopy.

## Data Availability

No new data were created or analyzed in this study. Data sharing is not applicable to this article.

## Supplementary Materials

The following supporting information can be downloaded at: https://www.mdpi.com/article/10.3390/diagnostics16040635/s1, Table S1. Animal/in vivo experimental studies (2000–present). And Table S2. In vitro and experimental studies investigating metal release into artificial saliva or saliva-simulating media (2000–present).

## Abbreviations

The following abbreviations are used in this manuscript:

AAS	Atomic Absorption Spectrometry
AI	Artificial Intelligence
Al	Aluminum
ATP	Adenosine Triphosphate
Sb	Antimony
As	Arsenic
Cd	Cadmium
Co	Cobalt
Cr	Chromium
Cu	Copper
CV-AAS	Cold Vapor Atomic Absorption Spectrometry
DNA	Deoxyribonucleic Acid
EMT	Epithelial–Mesenchymal Transition
Fe	Iron
GFAAS	Graphite Furnace Atomic Absorption Spectrometry
Hg	Mercury
HPLC–ICP-MS	High-Performance Liquid Chromatography coupled with Inductively Coupled Plasma Mass Spectrometry
ICP-MS	Inductively Coupled Plasma Mass Spectrometry
ICP–OES	Inductively Coupled Plasma Optical Emission Spectrometry
IL	Interleukin
IARC	International Agency for Research on Cancer
IoMT	Internet-of-Medical-Things
MDA	Malondialdehyde
miRNA	MicroRNA
ML	Machine learning
Mn	Manganese
mtDNA	Mitochondrial DNA
NF-κB	Nuclear Factor kappa-light-chain-enhancer of activated B cells
Ni	Nickel
NIST	National Institute of Standards and Technology
Nrf2	Nuclear factor erythroid 2–related factor 2
OSCC	Oral Squamous Cell Carcinoma
Pb	Lead
POC	point-of-care
ppb	Parts per Billion
ROS	Reactive Oxygen Species
SERS	surface-enhanced Raman spectroscopy
sIgA	Secretory Immunoglobulin A
STAT3	Signal Transducer and Activator of Transcription 3
Ti	Titanium
TLR	Toll-Like Receptor
TNF-α	Tumor Necrosis Factor Alpha
Zn	Zinc
8-OHdG	8-hydroxy-2′-deoxyguanosine
