# Supplementary material for: Toward Smart Salivary Diagnostics: A Comprehensive Review of Heavy Metal Biomarkers and Digital Risk Modeling
Source: Diagnostics (Basel). 2026 Feb 22;16(4):635. doi: 10.3390/diagnostics16040635 (PMC12939596; doi:10.3390/diagnostics16040635)
Supplement: Supplementary file 1 [file diagnostics-16-00635-s001.zip › diagnostics-4171602-supplementary.pdf]

Supplementary Table S1. Animal / *in vivo* experimental studies (2000–present).

Study / Year	Model	Analytical Method	Metals Assessed	Biological matrix	Main Findings
Abdollahi *et al*., 2000 [175]	Rat	-	Cd	Saliva flow	Significant alteration of salivary glands, due to systemic exposure
Abdollahi *et al*., 2003 [176]	Rat	-	Cd	Salivary glands	Oxidative stress in salivary glands, due to systemic exposure
Timchalk *et al*., 2006[177]	Rat	AAS	Pb	Saliva, blood	Demonstrates saliva as Pb excretion route. Rapid detection
Kostecka-Sochoń *et al*., 2018 [178]	Rat	-	Cd	Salivary glands	Oxidative stress in salivary glands, due to systemic exposure
Souza-Monteiro *et al*., 2022 [52]	Rat	ICP-MS/ proteomics	Al	Salivary glands	Biological plausibility for Al-related salivary dysfunction
El-Fatah *et al*., 2024 [53]	Rat	-	Cd	Salivary glands	Salivary gland alteration

Abbreviations: AAS, atomic absorption spectrometry; ICP-MS, inductively coupled plasma mass spectrometry.

Note: Animal studies often measure gland accumulation rather than saliva itself; these support mechanistic validity for salivary diagnostics.

Supplementary Table S2. *In vitro* and experimental studies investigating metal release into artificial saliva or saliva-simulating media (2000–present).

Study / Year	Model	Analytical Method	Metals Assessed	Biological matrix	Main Findings
Mikulewicz *et al*., 2011 [179]	Orthodontic appliances containing iron, chromium, nickel, silicon, and molybdenum	ICP-MS; SEM-EDS surface analysis	Mg, Al, Si, P, S, K, Ca, Ti, V, Mn, Fe, Co, Cu, Zn, Ni, Cr	Artificial saliva	Concentrations of multiple metals, including Al, Ni, and Cr, were significantly higher in artificial saliva incubated with orthodontic brackets, bands, and wires, demonstrating measurable ion release under simulated oral conditions.
Milošev *et al*., 2013 [180]	Ti metal and Ti alloys samples	ICP-MS; electrochemical testing; surface analysis (XPS/SEM)	Ti, Al, V/Nb	Artificial saliva ± fluoride	Corrosion behavior of Ti metal and Ti alloys was strongly affected by fluoride ions, with increased corrosion activity and enhanced metal ion release in fluoride-containing artificial saliva.
Souza *et al*., 2015 [181]	Pure Ti and Ti alloys	Electrochemical methods; ICP-MS; surface characterization	Ti, Al, V	Fluoride-containing media	High release of metallic ions after immersion at high fluoride concentration was detected, that can be potentially toxic to oral tissues.
Wepner *et al*., 2021 [182]	Removable orthodontic appliances	ICP-MS	Al, Cr, Ni	ISO 10271 corrosive medium (saliva-relevant)	Mechanical loading and the presence of glitter particles in orthodontic resin materials significantly increased metal ion release, particularly Al.
Leban *et al*., 2022 [183]	Additively manufactured (SLM) and wrought Ti-alloy samples	Electrochemical corrosion testing; ion release analysis; surface characterization	Ti, Al, V	Artificial saliva; fluoride and acidified media	Higher ion release was observed in fluoride-containing and acidified media, with additively manufactured (SLM) specimens releasing more ions than wrought Ti alloys.
Haleem *et al*., 2023 [184]	Orthodontic braces	ICP-AES	Pb, Al, Cr, Cu, Ba (plus Na, Li, K, Mg, Ca)	Artificial saliva	Artificial saliva pH increased after incubation. Ten ions, including Pb and Al, were detected, confirming release of heavy and trace metals from orthodontic braces.
Didović *et al*., 2025 [185]	NiTi and stainless steel orthodontic appliances	ICP-MS; AFM; SEM-EDX; XPS	Al, Fe, Cu, Ni, Cr	Artificial saliva and proteinaceous	Protein-containing media promoted the release of Fe, Cu, and Al, while suppressing Ni and Cr, highlighting the influence of biological components on metal release profiles.

Abbreviations: SLM, selective laser melting; SEM, scanning electron microscopy; EDS/EDX, energy-dispersive X-ray spectroscopy; XPS, X-ray photoelectron spectroscopy; AFM, atomic force microscopy; ICP-AES, inductively coupled plasma atomic emission spectrometry; NiTi, nickel–titanium alloy; ISO, International Organization for Standardization.
